# Additive Manufacturing of Smart Footwear Components for Healthcare Applications

**DOI:** 10.3390/mi16010030

**Published:** 2024-12-28

**Authors:** Aravind Kanna Kundumani Janarthanan, Bala Vaidhyanathan

**Affiliations:** Department of Materials, Loughborough University, Loughborough LE11 3TU, UK

**Keywords:** additive manufacturing, 3D printing, stereolithography, lattices, footwear, plantar pressure, foot care, flexible material

## Abstract

Diabetic foot complications pose significant health risks, necessitating innovative approaches in orthotic design. This study explores the potential of additive manufacturing in producing functional footwear components with lattice-based structures for diabetic foot orthoses. Five distinct lattice structures (gyroid, diamond, Schwarz P, Split P, and honeycomb) were designed and fabricated using stereolithography (SLA) with varying strand thicknesses and resin types. Mechanical testing revealed that the Schwarz P lattice exhibited superior compressive strength, particularly when fabricated with flexible resin. Porosity analysis demonstrated significant variations across structures, with the gyroid showing the most pronounced changes with increasing mesh thickness. Real-time pressure distribution mapping, achieved through integrated force-sensitive resistors and Arduino-based data acquisition, enabled the visualization of pressure hotspots across the insole. The correlation between lattice properties and pressure distribution was established, allowing for tailored designs that effectively alleviated high-pressure areas. This study demonstrates the feasibility of creating highly personalized orthotic solutions for diabetic patients using additive manufacturing, offering a promising approach to reducing the plantar pressure in foot and may contribute to improved outcomes in diabetic foot care.

## 1. Introduction

Foot-related disorders are known to be one of the prominent root causes for the deterioration of human quality of life, as they are closely associated with mobility impairment. Abnormal foot postures during gait can often lead to pain and lower-extremity pathologies. Foot pain and related disabilities have been increasingly recognized in recent years, with prevalence ranging from 13% to 36% in population cohorts aged from 20 to 44 years, and these rates tend to increase with age [1]. The Centers for Disease Control and Prevention (CDC) estimates that approximately one-third of older adults have diabetes, which increases the risk of complications compared with younger adults with diabetes. The projected growth of this demographic is apparent with the NHS in England revealing that there are nearly 35 million registered cases of diabetes [2]. Foot ulcers are common in patients with diabetes, with one in 50 patients with diabetes affecting every year. They also have much higher risk of ulcer and amputation than non-diabetics, with one diabetic patient amputated every 30 s in the world. The prevalence of ulcer increases, and so does the risk of major amputations in relation to diabetes, with increasing age. Effective prevention of foot ulcers relies on the identification of high-risk patients and the management of individual and general risk factors including inappropriate footwear, barefoot walking and lack of self-care [3]. One possible solution includes custom-made shoes; however, the concept of customization needs to be amended to entail varying degrees of consumer customizations from design, size, fit, and pertaining to the patient themselves [4].

With advancements in manufacturing technology, personalization in healthcare is attracting considerable attention. One of the biggest breakthroughs in this domain is additive manufacturing (AM), more popularly known as 3D printing. AM can create complex structures that are impossible to produce using conventional methods. This technology has been applied in various disciplines, including aerospace, techniques for manufacturing lattice-based structures with favorable mechanical properties for tissue engineering, among others [5].

Lattice structures have different structural design challenges which need to be solved by optimizing not just the size, but also shape and topology design methods. Topology optimization is concerned with support and boundary conditions with respect to the spatial arrangement within the structure [6]. Various cellular lattice structures, produced in other configurations or geometries, have received much attention and research to improve the mechanical performance [7,8,9]. In recent years, there has been a move towards additive shoe manufacturing, where changeable designs have enabled manufacturers to customize products [10].

Recent advances in insole design to reduce plantar pressure have focused on customization and innovative materials. Custom insoles designed using foot pressure mapping have been shown to be highly effective in reducing plantar pressure in diabetic patients and can reduce peak plantar pressure by 30–50%. These insoles typically use a multi-layer design to increase the contact area and distribute pressure evenly [11]. Material choices have evolved to include softer, more shock-absorbing compounds such as EVA (Ethylene Vinyl Acetate) and silicone-based materials. These materials have proven to be particularly effective in reducing plantar pressure, especially in high-risk areas such as the forefoot and heel [12]. Advances in technology have made it possible to integrate pressure mapping technology into insole designs, allowing for precise targeted relief of ulcer-prone areas. Studies have shown that effective insoles can reduce pressure below a critical value of 200 kPa, significantly reducing the risk of ulcers in diabetic patients [13].

This study intends to investigate the possible application of additive manufacturing in the fabrication of functional footwear elements, utilizing lattice-based forms of lattices to provide greater support and adjust to properties in a foot orthoses. We will assess the extent to which AM techniques enable the rapid and economic realization of smart footwear components that incorporate embedded functional materials that respond to stimuli from the external environment. We will examine SLA 3D printing technique for specific applications and create custom-fit footwear for foot deformities, such as those seen in diabetic patients, ultimately benefitting the patients further and also driving sustainable development of the production of footwear.

## 2. Materials and Methods

### 2.1. Design of Lattice Structures

Designing lattice structures involved a combination of computational modeling and experimental validation. Five distinct lattice structures were designed in this study using the parametric open-source modelling software (MSLattice) [14], Rhino 3D (Rhinoceros 3D, Version 6 SR22, Robert McNeel & Associates, Seattle, WA, USA), and nTopology (nTop Edu, Release 3.26, nTop Inc., New York, NY, USA): gyroid, diamond, Schwarz P, Split P, and honeycomb. Table 1 represents the equations of the Triply Periodic Minimal Structures (TPMS) [14,15,16].

**Figure 1:**
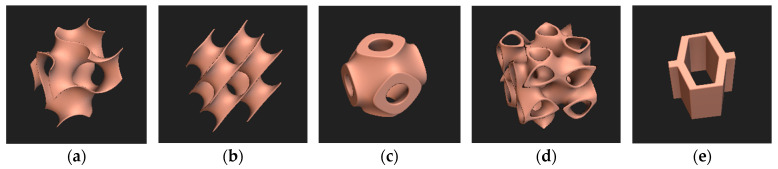
Unit cell structure preview of lattices (**a**) gyroid, (**b**) diamond, (**c**) Schwarz P, (**d**) Split P, and (**e**) honeycomb.

These structures were chosen to represent a range of geometries with varying mechanical properties and potential applications in footwear components. All structures were designed as cylinders with a diameter of 29 mm and a thickness of 12.5 mm, selected to conform with mechanical testing standards for rubber like materials. Figure 1 represents the unit cell structure for each lattice and the unit cell size for each structure was fixed at 5 × 5 × 5 mm.

The strand thickness of each lattice structure was varied to investigate the effect of structural density on mechanical properties. Three thickness values 0.5 mm, 0.75 mm and 1 mm were set on each lattice structure. The design process involved creating the basic unit cell for each structure type and then replicating it to fill the cylindrical volume. Each structure was meshed using triangular elements with a tolerance of 0.1 mm to capture the intricate geometries of the lattice structures, particularly for the thinner strands. The meshed models were then exported as STL (stereolithography) files, a format compatible with most 3D printing systems.

### 2.2. D Printer and Material Selection

In this study, the Form 2 SLA printer (Formlabs, Somerville, MA, USA) was utilized to fabricate the designed lattice structure. Two proprietary materials from Formlabs were selected for their specific mechanical properties: Formlab Elastic Resin and Formlabs Flexible Resin. These materials were chosen to match the elastomeric properties for footwear applications. Further specifications of the printer and hardness property of the materials are summarized in Table 2.

The STL files of the designed lattice structures were imported into PreForm (Release 3.21.0), Formlabs slicer software. Within PreForm, the models were centered on the build platform and print parameters were adjusted to optimize the balance between print quality, speed, and material usage for each resin type. The layer thickness of the prints was set to 100 μM. Following the completion of the printing process, parts were carefully removed from the build platform and preformed an ultrasonic rinse in an isopropyl alcohol (IPA) bath for 10 min. After rinsing, the parts were thoroughly dried using compressed air for removal of IPA residues. The post-processing involved UV curing which was performed using a FormCure (Formlabs, Somerville, MA, USA) for 20 min at 60 °C to achieve optimal mechanical properties and ensure complete polymerization of the resin.

### 2.3. Characterization and Analysis of Printed Structures

After post-processing and drying, samples were measured with Mitutoyo 150 mm Digital Caliper (Metric, Absolute) and weighed on a high-precision balance (1 μg sensitivity) to ensure accurate density assessment of the printed structures. For microstructural characterization, a Zeiss Primotech Microscope (Carl Zeiss Limited, Cambridge, UK) was utilized with a 7.5× optical zoom. Microscopic images of individual strands within the structures were captured at 500 μM magnification using Carl Zeiss Matscope software.

The porosity of the lattice structures was quantified through experimental measurements. The void fraction was calculated using the following equation:

(1) $$\mathrm{Void}\;\mathrm{fraction}=\left[\frac{V_{part\;}-V_{resin}}{V_{part}}\right]\times 100$$

where

*V_part_* is the total volume of the lattice structure sample (mm^3^), and

*V_resin_* is the volume of resin material in the lattice structure (mm^3^).

The total volume (*V_part_*) was calculated from the dimensions of the cylindrical samples (29 mm diameter, 12.5 mm thickness) using the formula for cylinder volume:

V = ∏r^2^ h (2)

The volume of resin (*V_resin_*) was determined by weighing the printed and post-processed sample on a high-precision balance (1 μg sensitivity) and dividing the mass by the known density of the cured resin material.

Compression tests were conducted using a Tinius Olsen Universal Testing Machine with a 1 KN load capacity, following ASTM standard D575-91 for compressive properties of rubber [17]. According to the ASTM standard, two cylindrical samples (29 mm diameter, 12.5 mm thickness) of each type and strand thickness were tested.

### 2.4. Sensor Integration and Pressure Analysis

**Figure 2:**
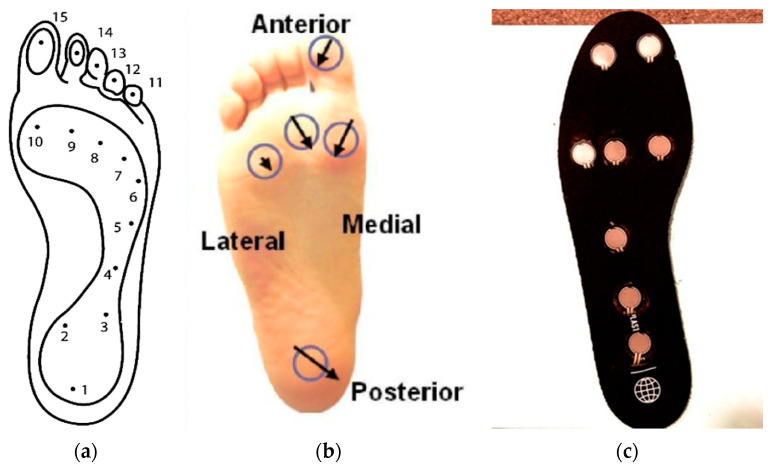
Sensor placement system: (**a**) anatomical foot with pressure points [21], (**b**) regional resultant shear in balanced standing [22], and (**c**) placement of FSR.

Force Resistive Sensors, FSR 402 (Interlink Electronics) were placed on the commercially available insole at locations corresponding to peak pressure areas, as identified through the literature [18,19,20]. The active area of each FSR sensor was carefully affixed to the contact regions of the insole, while the wiring was discreetly routed through the slits at the back to avoid direct contact with the foot. Figure 2 shows the placement of sensor based on the plantar points on the foot. The sensors were positioned at the heel, first and fifth metatarsal heads, and hallux, based on established literature on plantar pressure distribution in diabetic feet. The sensors were connected to an Arduino nano 33 BLE sense microcontroller (Arduino) for data acquisition and processing. Arduino IDE (Arduino, Release 2.2.1) was programmed to receive force data from the sensors and output analog values via the serial monitor at a rate of 9600 baud per second. Further specifications of the FSR 402 and Arduino used in this study are summarized in Table 3. The analog output ranged from 0 (indicating no pressure) to 1023 (representing maximum pressure), allowing precise monitoring of pressure variations across specific points on the insole.

Further, a pressure mapping technique was incorporated in design process. The force data obtained from the Arduino serial monitor were captured and transformed into a color map using MATLAB (The MathWorks Inc., Natick, MA, USA, MATLAB version R2022a) and imported as a .csv file into design software. Utilizing the pressure map, the thickness of the lattice structure was adjusted within a range of 0.5 mm to 1.2 mm, based on the pressure distribution gradients established by a color code scale from 0 to 255. Finally, real-time testing was performed to investigate the inverse pressure analysis.

## 3. Results

### 3.1. Mechanical Properties of Lattice Structures

#### 3.1.1. Compressive Strength

**Figure 3:**
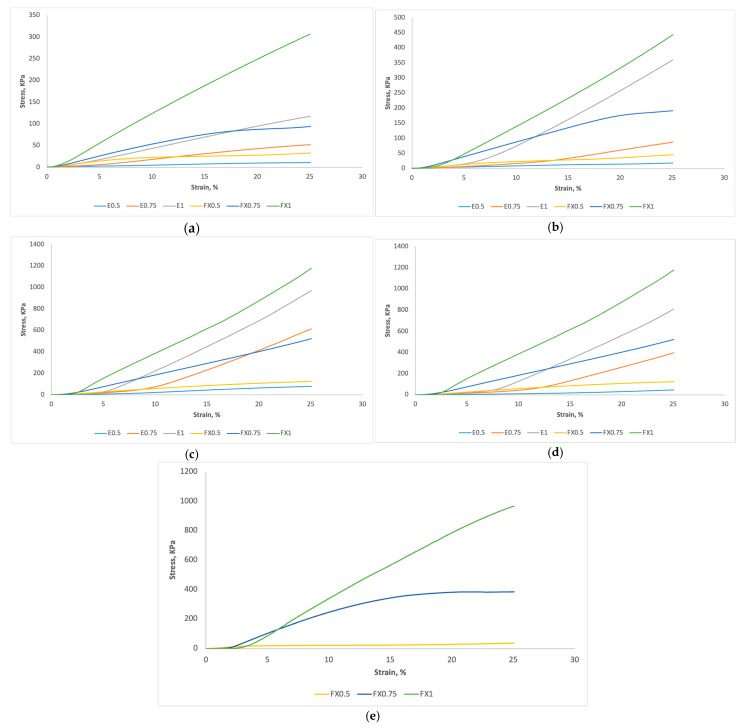
Stress–strain curves of different lattice structures with strand thickness of 0.5 mm, 0.75 mm and 1 mm for elastic and flexible material (**a**) gyroid, (**b**) diamond, (**c**) Schwarz P, (**d**) Split P, and (**e**) honeycomb.

The mechanical properties of Triply Periodic Minimal Surface (TPMS) structures and simple lattice structures were evaluated through axial compression tests. Stress–strain curves were generated for structures with mesh thicknesses of 0.5 mm, 0.75 mm, and 1 mm, fabricated using both elastic (E) and flexible (FX) resin materials shown in Figure 3.

For the gyroid structure (Figure 3a), the highest compressive strength was observed in FX1 (flexible resin, 1 mm thickness) at 307 kPa, while E0.5 (elastic resin, 0.5 mm thickness) exhibited the lowest strength at approximately 10 kPa. All curves demonstrated an elastic limit at 25% compression and displayed isotropic characteristics.

The diamond (Figure 3b)and Schwarz P (Figure 3c) structures showed similar trends to the gyroid structure, with flexible resin consistently yielding higher compressive stress compared to elastic resin. In the diamond lattice, FX1 exhibited the highest compressive stress, while E0.5 recorded the lowest.

The Schwarz P lattice emerged as the strongest configuration, with FX1 achieving a compressive strength of 1200 kPa, the highest among all tested structures and the Split P lattice (Figure 3d), reaching a maximum compressive stress of 820 kPa.

Due to printing challenges, the honeycomb lattice (Figure 3e), was only printable with flexible resin, achieving a compressive strength of 980 kPa.

Across all structures, increasing strand thickness from 0.5 mm to 1 mm resulted in higher compressive strengths. This trend was consistent for both elastic and flexible resins, though more distinct in the flexible resin samples. Among the TPMS structures, the Schwarz P lattice demonstrated the highest compressive strength, followed by the diamond and gyroid structures. The simple lattice structures (honeycomb) showed competitive strength, but their limited printability with certain resins restricted comprehensive comparison.

In all comparable cases, structures fabricated with flexible resin exhibited higher compressive strengths than those made with elastic resin. This trend was consistent across different lattice geometries and strand thicknesses, suggesting that the flexible resin’s properties are more conducive to load-bearing applications in these lattice structures.

#### 3.1.2. Specific Strength Analysis

**Figure 4:**
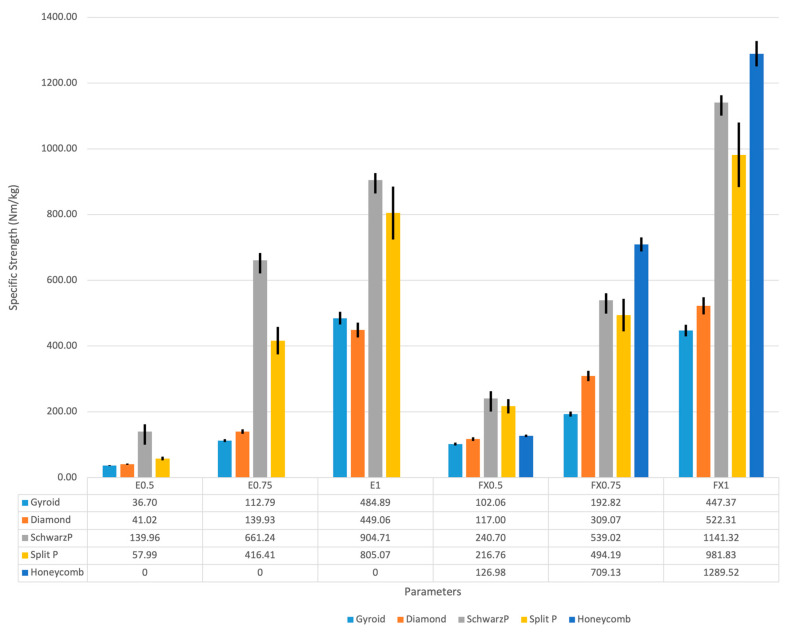
Specific strength variations across different lattice structures.

To evaluate the efficiency of the lattice structures in terms of their strength relative to weight, specific strength was calculated for each configuration. Figure 4 presents the specific strength variations across different lattice structures fabricated using flexible and elastic resins.

The honeycomb lattice demonstrated the highest specific strength, particularly with a 1 mm mesh thickness. This result suggests that the honeycomb structure offers an efficient combination of strength and lightweight properties. Among the structures fabricated with elastic resin, the Schwarz P lattice exhibited the highest specific strength, likely due to its complex design and increased mesh thickness. The gyroid lattice structure, while showing lower specific strength values overall, displayed a consistent increase in specific strength with increasing mesh thickness. This trend was observed for both flexible and elastic resin formulations.

Strand thickness emerged as a critical factor influencing specific strength, especially in structures fabricated with flexible resin. A positive correlation between strand thickness and specific strength was evident across all lattice types.

The specific strength analysis revealed distinct performance characteristics for each lattice structure:

1. Honeycomb: Exhibited the highest specific strength, particularly at 1 mm mesh thickness.
2. Schwarz P: Demonstrated superior specific strength among elastic resin structures.
3. Gyroid: Showed a consistent, incremental increase in specific strength with mesh thickness.
4. Diamond and Split P: Displayed intermediate specific strength values, with performance varying based on resin type and mesh thickness.

### 3.2. Structural Characterization

#### 3.2.1. Porosity Analysis

**Figure 5:**
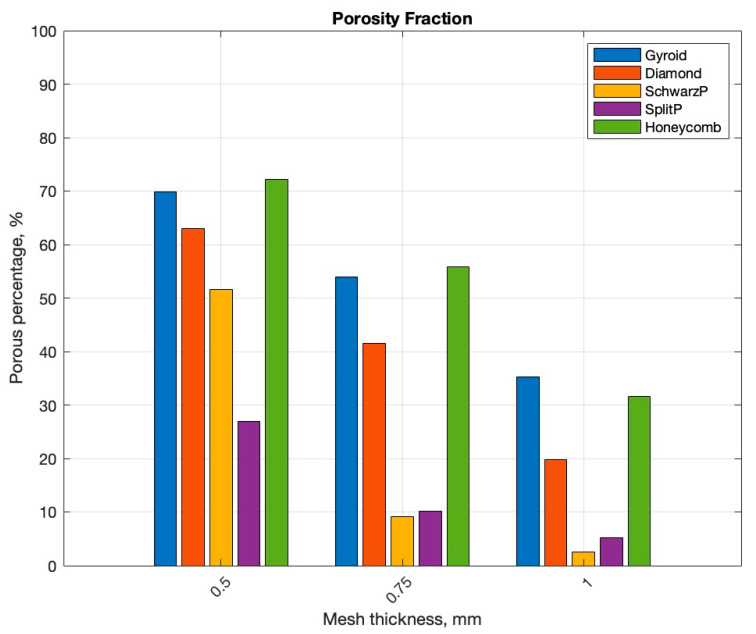
Porosity comparison of lattices with different strand thickness.

The porosity of the lattice structures was analyzed as a function of mesh thickness. Figure 5. illustrates the porosity variations for each lattice type across different mesh thicknesses. The gyroid lattice structure exhibited a significant decrease in porosity with increasing mesh thickness. At the lowest thickness (0.5 mm), it demonstrated the highest initial porosity among all structures tested. However, this porosity rapidly declined as the mesh thickness increased to 1 mm. The diamond structure maintained a more consistent porosity across the range of mesh thicknesses examined. While its initial porosity at 0.5 mm thickness was lower than that of the gyroid, it showed less variation as thickness increased to 1 mm.

The Schwarz P structure displayed a substantial reduction in porosity as mesh thickness increased. This decline was more pronounced compared to the diamond structure but less severe than the gyroid. The Split P structure exhibited the least variation in porosity across different mesh thicknesses. Its porosity remained relatively stable from 0.5 mm to 1 mm thickness. The honeycomb structure showed a moderate decrease in porosity with increasing mesh thickness. Its porosity reduction was less pronounced than the gyroid and Schwarz P structures but more significant than the Split P.

#### 3.2.2. Microscopic Analysis

Microscopic analysis was conducted on fabricated samples with varying strand thicknesses to characterize the impact of the printing process on strut quality. The investigation focused on both the top and bottom layers of the samples, as no support structures were used during printing.

**Figure 6:**
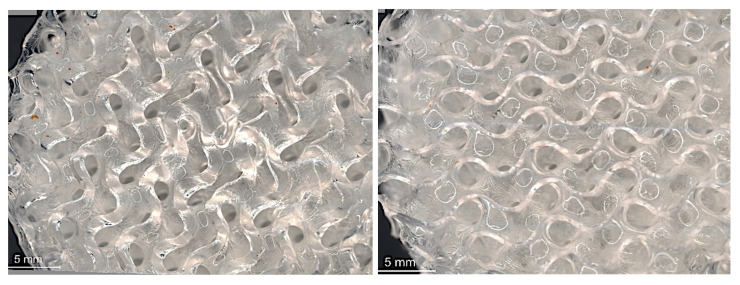
Microscopic image of gyroid (0.5 mm strand thickness)—elastic material: bottom layer (**left**) and top layer (**right**).

Top Layer Analysis:

Examination of the top layers revealed distinct patterns corresponding to the chosen strand thicknesses. For the gyroid lattice structure with 0.5 mm strand thickness fabricated using elastic resin (Figure 6), the top layer exhibited precise geometrical features with no visible breakages. The circular marks observed were attributed to light reflection rather than printing defects.

Bottom Layer Analysis:

The bottom layers, representing the initial printed layer in contact with the build platform, provided insights into adhesion and stability during the printing process. For the gyroid structure with 0.5 mm strand thickness using elastic resin, a difference in layer thickness between the top and bottom layers was observed. This variation was attributed to challenges with initial layer adhesion, resulting in overcuring of the first layer.

**Figure 7:**
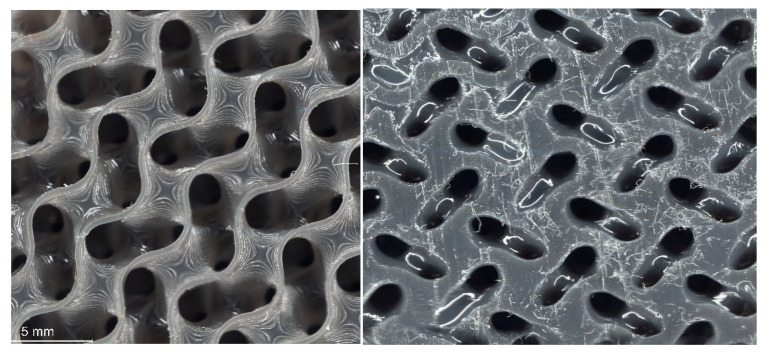
Microscopic image of gyroid (0.5 mm strand thickness)—flexible material: bottom layer (**left**) and top layer (**right**).

When comparing the gyroid structure (0.5 mm strand thickness) fabricated with flexible resin shown in Figure 7. to that made with elastic resin, several differences were noted:

1. The flexible resin samples showed more distinct overcuring in the initial layer, leading to increased wall thickness compared to the elastic resin samples.
2. Resin residues were observed on the bottom layer of the flexible resin samples. This was likely due to cell size reduction from overcuring, which hindered resin flow and led to residue accumulation.
3. Despite the variations in the bottom layer, the top layer of the flexible resin samples maintained superior quality with precise geometrical features and no breakages.

### 3.3. Sensor Integration and Pressure Analysis

#### 3.3.1. Pressure Distribution Mapping

The pressure distribution across the insole was successfully visualized using an integrated sensor system and data processing methodology. Eight pressure sensors, interfaced with an Arduino microcontroller, captured real-time pressure values at key locations on the insole.

**Figure 8:**
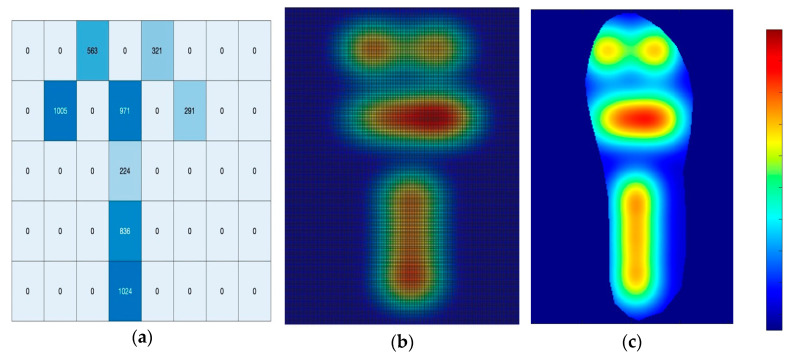
Pressure distribution mapping process: (**a**) visualization of monochrome map in the matrix, (**b**) color map using Gaussian smoothening, and (**c**) Gaussian smoothening encapsulated within the insole.

Figure 8a illustrates the initial heatmap visualization of the pressure data in matrix form. This representation provides a discrete view of pressure values at the sensor locations, offering a foundational understanding of pressure distribution patterns. To enhance the visual representation and interpolate between sensor points, a Gaussian smoothing algorithm was applied to the raw data.

Figure 8b demonstrates the resulting smoothed heatmap, which provides a more continuous and visually coherent representation of the pressure distribution across the insole surface. Further refinement of the pressure map was achieved by applying an insole-shaped mask to the smoothed data. Figure 8c shows the final pressure distribution map, where the heatmap is confined within the anatomical contours of the insole. This representation allows for a more accurate interpretation of pressure patterns in relation to foot anatomy.

The pressure intensity is represented by a color gradient, with warmer colors (reds and yellows) indicating areas of higher pressure, and cooler colors (greens and blues) representing areas of lower pressure. The heatmap reveals distinct calibrated pressure concentrations in the heel and forefoot regions, with relatively lower pressure observed in the midfoot area to obtain the accurate results in real-time testing.

During pressure analysis, subjects stood relaxed on two feet with their feet shoulder-width apart, a posture commonly used for static measurements of plantar pressure The pressure patterns we observed, showing peak pressures under the heel and metatarsal heads, are consistent with typical findings in diabetic foot studies [23].

Building upon the pressure distribution mapping results, a quantitative analysis of the force values was conducted corresponding to the Arduino analog data and their associated color codes. The Arduino analog data range of 0–1024 was calibrated to represent a force range of 0 to 10 kg, while the color code ranges from 0 to 255.

Table 4 presents the calculated pressure values for Arduino analog data in multiples of 100, ranging from 0 to 1000, along with their corresponding color codes and pressure values.

This calibration allows for a more precise interpretation of the pressure distribution visualized in the heatmap. The pressure values range from 0 kPa (no pressure) to 565 kPa (maximum pressure), providing a quantitative measure of the force exerted on different areas of the insole. The correlation between Arduino analog data, color codes, and pressure values enables a more detailed analysis of the pressure distribution across the foot. Areas displaying warmer colors in the heatmap correspond to higher pressure values, with the maximum pressure of 565 kPa potentially indicating areas of concern for individuals at risk of foot-related complications.

#### 3.3.2. Correlation Between the Lattice Structure and Pressure Distribution

**Figure 9:**
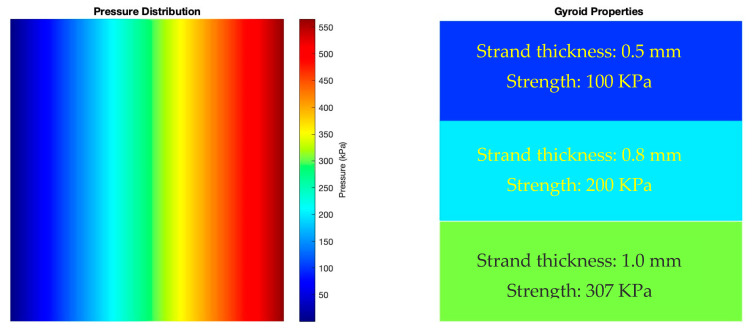
Correlation between the lattice structure and pressure distribution: pressure distribution with corresponding pressure values (**left panel**) and gyroid properties with corresponding color codes (**right panel**).

The gyroid structure was chosen due to its interconnected, self-supporting geometry and demonstrating a good balance between strength and flexibility. Figure 9 illustrates the correlation between gyroid lattice properties and pressure distribution across the insole. The left panel of the figure presents a color-coded pressure map, with cooler colors (blue) indicating lower pressures and warmer colors (red) at the bottom representing higher pressures. This gradient corresponds to the pressure values ranging from 0.56 kPa to 564.92 kPa, as measured by the sensor array. The right panel of Figure 9 depicts the gyroid lattice properties implemented in different regions of the insole. The gyroid structure, a Triply Periodic Minimal Surface (TPMS), was selected for its unique mechanical properties and potential for tailored performance. The insole is divided into three main regions: forefoot, midfoot, and heel, each with distinct gyroid characteristics.

In the region, where lower pressures were observed, a gyroid structure with a thickness of 0.5 mm was implemented. This thinner structure allows for greater flexibility in the dynamic forefoot area while providing adequate support for pressures of approximately 100 kPa. The region, experiencing intermediate pressures, utilized a gyroid structure with a thickness of 0.75 mm. This configuration offers a balance between support and flexibility, accommodating the transitional nature of this area and pressures approximately 200 kPa. The region, where the highest pressures were recorded (up to 564.92 kPa), employed a gyroid structure with a thickness of 1.2 mm. This thicker structure provides maximum support and pressure distribution in the high-impact area.

The correlation between the pressure distribution and gyroid properties demonstrates a tailored approach to insole design. Areas of higher pressure, as indicated by warmer colors in the pressure map, correspond to regions with thicker gyroid structures. Conversely, areas of lower pressure, represented by cooler colors, align with thinner gyroid structures.

### 3.4. Real-Time Data Analysis

**Figure 10:**
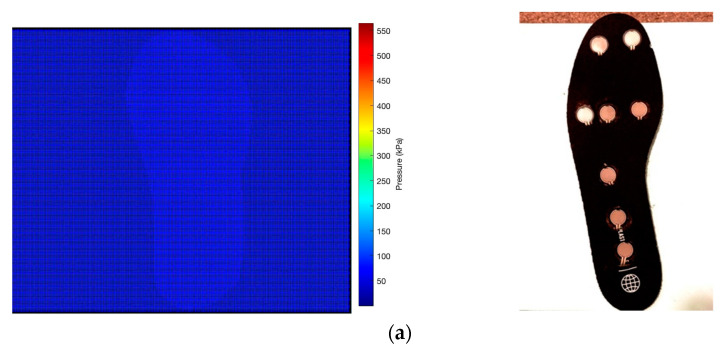

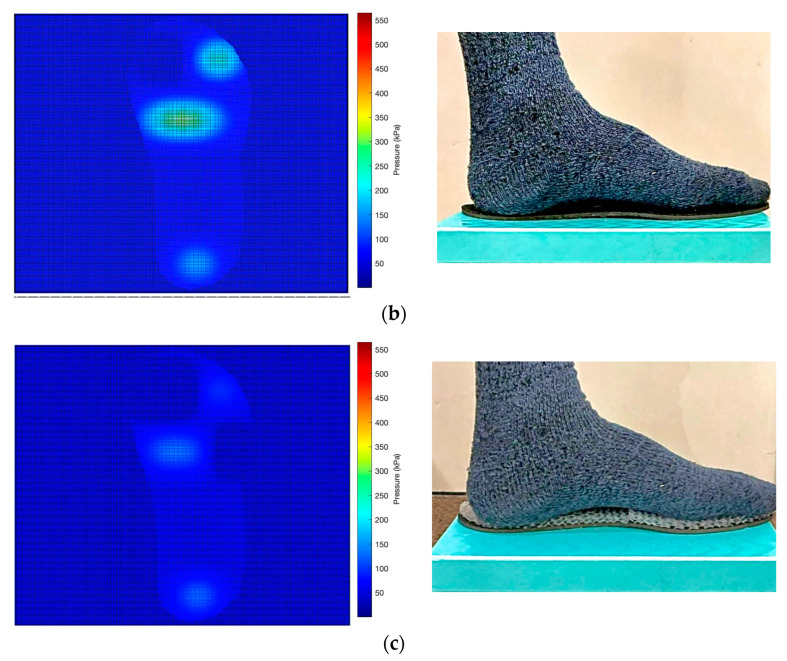
Real-time pressure distribution of right foot at different instances—pressure map (left) corresponding to the right foot placed on the insole (right)—(**a**) no pressure applied, (**b**) direct foot placement, and (**c**) foot placed on custom-designed insole (alleviating pressure hotspots is evident).

The real-time pressure analysis of the sensor-equipped insole provided valuable insights into the efficacy of the custom-designed structure. Figure 10 illustrates the pressure distribution at three distinct instances during the experimental procedure. Figure 10a depicts the baseline scenario with no pressure applied to the insole, serving as a control for subsequent measurements. Upon direct right foot placement (Figure 10b), the sensors captured the static pressure distribution, revealing areas of high-pressure concentration. These ’hotspots’ typically correspond to regions of the foot prone to ulceration in diabetic patients, such as the heel and metatarsal heads. The most significant findings emerged when the right foot was placed on the custom-designed insole (Figure 10c). The pressure map demonstrates a marked redistribution of forces, with a notable alleviation of pressure in previously identified hotspots. This reduction in localized high-pressure areas is attributed to the tailored structural modifications implemented in the insole design, particularly the strategic variation in gyroid lattice thickness.

The real-time data acquisition allowed for a dynamic assessment of the insole’s performance. As the foot interacted with the custom-designed surface, the sensors recorded shifts in pressure distribution that corresponded directly to the structural adaptations in the insole. Areas with thicker gyroid structures, designed to provide enhanced support, showed a more even distribution of pressure. Conversely, regions with thinner structures exhibited greater flexibility, conforming to the foot’s contours and reducing pressure peaks. The inverse pressure design approach, informed by initial pressure mapping, proved effective in mitigating areas of high pressure. This is evidenced by the more homogeneous pressure distribution observed in Figure 10c compared to the direct foot placement scenario in Figure 10b. The reduction in pressure hotspots suggests that the custom-designed insole successfully redistributes forces across a larger surface area, potentially reducing the risk of pressure-induced complications.

## 4. Discussion

The findings have significant implications for the use of additive manufacturing techniques to create functional footwear components with lattice structures, especially in terms of their incorporation into diabetic foot orthoses. The mechanical testing, structural characterization and pressure distribution studies completed here thus provide a wealth of information regarding the capabilities and suitability of multiple lattice structures in this regard.

Compressive strength testing indicated that there were notable differences between the various lattice structures and material compositions that were tested. The Schwarz P lattice at 1 mm thick and made of flexible resin always showed the highest compressive strength. This result is consistent with previous work showing the load bearing efficiency of TPMS structures [24]. Considering the excellent performance of the Schwarz P, it is possible that this type of lattice can be used to provide adequate support in high-pressure areas of diabetic foot orthoses, such as the heel region, the area that requires maximum support. The trend of higher compressive strength with increasing strand thickness across all structures is aligned with basic materials science and is consistent with previous research on lattice structures [25]. However, the significant variation in performance between flexible and elastic resins highlights the importance of material selection in achieving desirable mechanical qualities. This finding adds to our understanding of material-structure interactions in 3D-printed lattices and underlines the importance of taking geometry and material properties into account when designing orthotics. 

A specific strength analysis was performed to offer additional perspective of efficacy of various scaffolds. A higher specific strength would thus likely be observed with the honeycomb lattice structure, especially at the 1 mm mesh thickness considered, substantiating existing literature on the effectiveness of honeycomb structures in lightweight applications [26]. This feature would be particularly beneficial for development of lightweight but supportive orthotic devices for diabetic patients who require both pressure redistribution and ease of wear.

Porosity results also indicated different behavior with different lattices, as the gyroid structure in particular demonstrated the most significant porosity change with varying mesh thickness. This property could be used to create multi-gradient structures in a single orthotic that would provide customized support and cushioning throughout the foot. The microscopic analysis highlighted the challenges and considerations in the 3D printing process, particularly regarding initial layer adhesion and overcuring. These observations highlight the importance of optimizing printing parameters to ensure consistent structural integrity throughout the printed component, a crucial factor in the reliability and performance of orthotic devices.

The pressure distribution mapping demonstrated the effectiveness of the integrated sensor system in capturing real-time pressure data. This approach adds upon previous work in plantar pressure measurement [20] but extends it by incorporating direct feedback into the design process. The refined heatmap visualization provides a powerful tool for identifying pressure hotspots and enhancing the design of customized orthotic devices. The ability to correlate pressure distribution with lattice structure properties opens new opportunities for designing a personalized footwear components to meet individual patient needs. This approach aligns with the growing trend towards personalized medicine and could significantly improve the efficacy of diabetic foot care interventions [27].

The findings of this study have several important implications for the field of diabetic foot care and orthotic design:

1. Personalized Orthotic Solutions: The ability to adjust structural properties through lattice design and material selection offers a level of customization previously unachievable with traditional manufacturing methods. Future research should focus on development of algorithms that can automatically generate optimal lattice designs based on individual pressure profiles.
2. Dynamic Pressure Management: The real-time pressure sensing capabilities demonstrated in this study could be extended to enable dynamic orthotic systems that adapt to spatially varied pressure distributions during gait. This could involve the integration of smart materials or actuators into the lattice structures.
3. Long-Term Clinical Efficacy: Though initial data from this study suggest that individualized offloading devices can be used to prevent diabetic foot complications, further clinical studies should affirm that custom-made orthotic devices can translate to long-term prevention of diabetic foot complications. Studies can be conducted in the future to evaluate longevity of the Lattice structures over time and its impact on foot health outcomes.
4. Manufacturing Optimization: Further research is needed to optimize 3D printing for mass production of personalized orthopedic appliances. This includes refining of printing parameters for minimizing structural inconsistencies as well as the exploration of new materials that possess desirable mechanical properties along with biocompatibility and durability.
5. Integration of Additional Functionalities: In the future, these orthotic devices could incorporate additional sensors for monitoring temperature, humidity, or even biochemical markers. This multi-modal sensing approach could provide a better evaluation of foot health and early detection of potential complications.

This study represents a significant step towards the development of next-generation footwear components that can address the complex needs of diabetic patients. The integration of advanced manufacturing techniques, materials science, and biomechanical principles demonstrated here has the potential to revolutionize the field of orthotic design and eventually contribute to better diabetic foot care in future.

## Figures and Tables

**Table 1:** Mathematical equations of different TPMS types.

Type	Equation, F(x,y,z) = c
Gyroid	$\sin\left(x\right)\cos\left(y\right)+\sin\left(y\right)\cos\left(z\right)+\sin\left(z\right)\cos\left(x\right)$ = c
Diamond	$\sin\left(x\right)\sin\left(y\right)\sin\left(z\right)+\sin\left(x\right)\cos\left(y\right)\cos\left(z\right)+\cos\left(x\right)\sin\left(y\right)\cos\left(z\right)+cos(x)cos(y)sin(z)$ = c
Schwarz P	$\cos\left(x\right)+\cos\left(y\right)+cos(z)$ = c
Split P	$1.1\times \left[\sin\left(2x\right)\sin\left(z\right)\cos\left(y\right)+\sin\left(2y\right)\sin\left(x\right)\cos\left(z\right)+\sin\left(2z\right)\cos\left(x\right)\right]-0.2\times \left[\cos\left(2x\right)\cos\left(2y\right)\cos\left(2z\right)+\cos\left(2z\right)\cos\left(2x\right)\right]-0.4\times [\cos\left(2x\right)+\cos\left(2y\right)+\cos\left(2z\right)]$ = c

**Table 2:** Printer Specifications and materials used in this study.

Printer Specifications	Formlabs Flexible Resin
Printer Model—Formlabs Form 2	Shore Hardness–80A
Technology—SLA	
Laser Power—250 mW	**Formlabs Elastic Resin**
Wavelength—405 nm violet laser	Shore Hardness–50A
Operating Temperature—35 °C	

**Table 3:** Arduino and sensor specifications used in this study.

Sensor Specifications	Arduino Specifications
Sensor—FSR 402	Microcontroller—Arduino Nano 33 BLE sense
Dimension—17.47 mm * 18.3 mm	Operating Voltage—3.3 V
Sensing dimension—∅14.68 mm	Clock Speed—64 MHz
Response time—<1 ms	Analog Input Pins—8
Working temperature—−30~60 °C	Weight—5 g

**Table 4:** Values corresponding to Arduino analog data and color codes.

Arduino Analog Data	Color Code	Mass (kg)	Force (N)	Pressure (Kpa)
0	0	0.01 ± 0.07	0.10 ± 0.69	0.56 ± 4.05
100	25	0.98 ± 0.12	9.62 ± 1.14	56.85 ± 6.72
200	50	1.95 ± 0.05	19.14 ± 0.53	113.14 ± 3.13
300	75	2.93 ± 0.08	28.70 ± 0.77	169.61 ± 4.55
400	100	3.90 ± 0.04	38.25 ± 0.42	226.08 ± 2.46
500	125	4.87 ± 0.10	47.81 ± 0.93	282.55 ± 5.52
600	150	5.85 ± 0.05	57.36 ± 0.45	339.02 ± 2.64
700	175	6.82 ± 0.07	66.92± 0.69	395.50 ± 4.09
800	200	7.80 ± 0.09	76.47 ± 0.88	451.97 ± 5.21
900	225	8.77 ± 0.11	86.03 ± 1.07	508.44 ± 6.32
1000	250	9.74 ± 0.12	95.58 ± 1.14	564.92 ± 6.72

## Data Availability

The raw data supporting the conclusions of this article will be made available by the authors on request.
